# Preparation of NaA Zeolite Composite Polyacrylonitrile Membranes (TiO_2_-NaA@PANMs) Doped with TiO_2_ and Adsorption Study of Sr^2+^

**DOI:** 10.3390/ma18092151

**Published:** 2025-05-07

**Authors:** Yu Liu, Erna Wei, Riwen Ji, Kaituo Wang

**Affiliations:** 1 State Key Laboratory of Featured Metal Materials and Life-Cycle Safety for Composite Structures, MOE Key Laboratory of New Processing Technology for Nonferrous Metals and Materials, Guangxi Key Laboratory of Processing for Non-Ferrous Metals and Featured Materials, School of Resources, Environment and Materials, Guangxi University, Nanning 530004, China; 19378999608@163.com; 2 Guangxi Beitou Industrial Co., Ltd., Nanning 530004, China; weierna2025@163.com; 3 Guangxi Zhuang Autonomous Region Institute of Product Quality Inspection, Nanning 530007, China

**Keywords:** NaA, PAN, electrospinning, TiO_2_, Sr^2+^

## Abstract

As a rarefied metallic element, strontium (Sr) is susceptible to significant environmental radioactive contamination risks during industrial mining and refining processes. In this study, NaA molecular sieves were prepared by alkali excitation using synthetic powders, which were homogeneously blended with the polyacrylonitrile (PAN) matrix, and nanoscale TiO_2_ reinforcing phases were introduced. Finally, composite separation membranes (TiO_2_-NaA@PANMs) with stable adsorption properties were constructed by electrostatic spinning technology. The micro-morphology and interfacial properties were characterized by SEM, XRD, and FT-IR systems. The adsorption experiments demonstrated that the equilibrium adsorption capacity of the system for Sr^2+^ reached 55.00 mg/g at the optimized pH = 6.0, and the theoretical saturated adsorption capacity at 298 K was 80.89 mg/g. The isothermal process conformed to the Langmuir’s model of monomolecular layer adsorption, and the kinetic behavior followed the quasi-secondary kinetic equation. Following three cycles of regeneration by elution with a 0.3 mol/L sodium citrate solution, the membrane material exhibited 81.60% Sr^2+^ removal efficacy. The composite membrane passages exhibited remarkable potential for utilization in engineering applications involving the treatment of complex nuclear wastewater.

## 1. Introduction

With the rapid development of nuclear energy technology, the environmental pollution caused by radionuclides is becoming increasingly serious; radioactive strontium, which has a long half-life and high biotoxicity, especially poses a major threat to the ecosystem and human health [1]. Traditional water treatment technologies such as chemical precipitation [2], ion exchange [3], and reverse osmosis [4] often face bottlenecks such as low selectivity and poor material stability when applied to nuclear wastewater with high salinity and multi-ion competition. For example, the adsorption capacity of commercial ion exchange resins for Sr^2+^ decreases under seawater concentration, and the functional groups are easily degraded under irradiation; although reverse osmosis membranes can realize desalination, the retention rate of Sr^2+^ is limited by its hydration radius (0.412 nm) and the insufficient matching of membrane pore size. Therefore, the development of novel adsorbent materials that combine high selectivity, irradiation resistance, and engineering applicability has become a key challenge in the field of nuclear wastewater treatment.

In recent years, molecular sieve materials have received widespread attention for their regular pore structure and tunable ion exchange performance [5]. Among them, NaA molecular sieves (LTA-type) have an eight-membered ring pore (~0.41 nm) that is highly compatible with the hydration radius of Sr^2+^, which can theoretically realize selective adsorption by “size sieving” [6]. However, pure NaA molecular sieves are susceptible to pulverization and loss in dynamic water flow. Polyacrylonitrile (PAN) is an ideal carrier material due to its excellent film-forming properties and chemical stability [7]. However, its inherent rigid chain segments may lead to increased membrane brittleness, and the main chain is prone to break under γ-ray irradiation. This contradiction has prompted researchers to introduce a third component to synergistically enhance the comprehensive performance of the materials.

Titanium dioxide (TiO_2_) nanoparticles have proven applications in nuclear waste curing due to their excellent irradiation stability. It has been shown that TiO_2_ can trap free radicals and inhibit the oxidative degradation of polymer chains under irradiation conditions, while its surface hydroxyl groups can specifically coordinate with Sr^2+^. However, the agglomeration tendency of TiO_2_ nanoparticles blocks the molecular sieve pores and reduces the utilization of effective adsorption sites [8]. Achieving the microstructural compatibility among TiO_2_, NaA molecular sieves, and PAN has become a core scientific issue for the construction of high-performance composite membranes.

However, TiO_2_/polymer composites prepared by conventional physical blending methods often face the problem of stress concentration due to nanoparticle agglomeration, and the loss of functional components under long-term hydrodynamic scouring. This phenomenon highlights the key shortcoming of traditional composite technology: the lack of chemical bonding between the inorganic and organic phases, which makes it difficult to achieve stable interfacial coupling at the nanoscale. In addition, most of the existing studies focus on single function enhancement (only enhancing irradiation stability or adsorption capacity), neglecting the multicomponent synergism on the comprehensive performance of materials.

The most commonly utilized methodologies for the preparation of membrane materials encompass phase separation [9,10], template synthesis [11,12], self-assembly [13,14], and electrostatic spinning [15,16]. In comparison to other methods, the electrostatic spinning method is characterized by reliable and precise technology, straightforward equipment requirements, and an accessible operational procedure. The method is employed in a multitude of fields, including cultural relics protection [17], battery production [18,19], catalytic decomposition [20,21], healthcare [22,23], food preservation [24,25], aerospace [26,27], and adsorption separation [28,29]. Additionally, the electrostatic spinning method can produce fibers with an ultrafine structure, resulting in nanomaterials with a higher specific surface area and porosity [30]. Additionally, the inherent physical and chemical properties of the fibers can be effectively preserved through this method, rendering it particularly advantageous for adsorption and filtration applications. The integration with adsorbents demonstrates excellent compatibility, ensuring synergistic enhancement of functional performance while maintaining structural integrity during operational processes.

Based on the above challenges, this study innovatively proposes the strategy of “electrostatic spinning-induced multistage assembly”: the formation of ultrafine fibers by a Taylor cone driven by a high-voltage electrostatic field, the ultra-high tensile rate of the polymer jet during electrostatic spinning to inhibit the sedimentation and aggregation of the molecular sieve particles, and the NaA molecular sieve TiO_2_ nanoparticles were uniformly distributed during the fiber curing process. Among them, PAN was used as the flexible matrix to construct a three-dimensional network structure by electrostatic spinning, which endowed the material with excellent mechanical toughness; NaA molecular sieves were embedded inside the PAN fibers, which used their pore size effect to achieve accurate sieving of Sr^2+^; and TiO_2_ nanoparticles were introduced to enhance the stability of the material. After the composite of the three, the effect of titanium dioxide addition on the adsorption performance of the membrane material was systematically investigated, and the preparation process and adsorption mechanism were explained by combining the analytical characterization means such as SEM, XRD, and FT-IR, etc. This breakthrough provides an innovative solution for the efficient removal of radioactive strontium through the composite of different materials.

## 2. Experimental Section

### 2.1. Materials

Dimethylformamide (Analytical Reagent (AR), ≥99.5%), sodium chloride (AR, ≥99.5%), anhydrous magnesium chloride (AR, ≥98.0%), anhydrous calcium chloride (AR, ≥96.0%), potassium chloride (AR, ≥99.5%), aluminum nitrate nonahydrate (AR, ≥99.5%), anhydrous ethanol (AR, ≥99.5%), sodium hydroxide (AR, ≥96.0%) were purchased from Guangdong Guanghua Technology Co. (Guangzhou, China); the polyacrylonitrile (PAN, molecular weight 150,000) used in the experiment was purchased from DuPont (Wilmington, DE, USA); the hydrochloric acid (AR, 36–38%) used in the experiment was purchased from Chengdu Cologne Chemical Co., Ltd. (Chengdu, China), and the standard solutions of sodium, magnesium, potassium, calcium, and strontium used in the experiment were purchased from the National Center for Analysis and Testing of Nonferrous Metals and Electronic Materials (Beijing, China). The tetraethyl orthosilicate (AR, ≥28.4% as SiO_2_), trisodium citrate dihydrate (AR, ≥99.0%), and strontium chloride hexahydrate (AR, 99.0–101.0%) used in the experiments were purchased from Sinopharm Chemical Reagent Co. (Shanghai, China). The surface water and lake water used in the experiments were obtained from Guangxi University. The seawater used in the experiments was obtained from Beihai, Guangxi.

### 2.2. Preparation of NaA@PANMs

**Figure 1:**
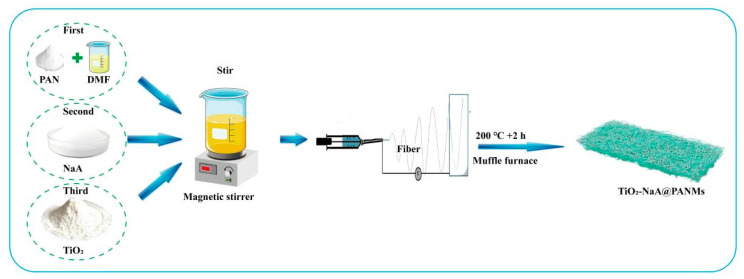
Experimental process for the preparation of TiO_2_-NaA@PANMs.

NaA molecular sieves were prepared and synthesized according to the literature previously reported by our group [31]. After undergoing a thorough washing and drying process, the prepared NaA molecular sieves were subjected to ball milling for a period of 6 h. This procedure was undertaken to achieve the formation of NaA powders of a smaller size, which were subsequently utilized. The preparation flowchart is shown in Figure 1. Polyacrylonitrile (PAN) was dissolved in an N, N-dimethylformamide (DMF) solution in a beaker. The beaker was placed in a 60 °C water bath, and the contents were stirred and heated for 2 h until the PAN was completely dissolved. This resulted in a PAN mass fraction of 10% of the solution. The beaker was then removed and placed on a magnetic stirrer, where it was added to a solution of PAN at a mass ratio of 100%, as well as to solutions of NaA molecular sieve powder at mass ratios of 2.5%, 5%, 7.5%, and 10% of nanometer-sized titanium dioxide. These solutions were stirred for a period of 12 h to ensure the formation of a uniform mixture. Ultrasonication was then performed for a duration of 10 min to remove any air bubbles that may have been present. These solutions were subsequently utilized for direct electrostatic spinning, resulting in a liquid solution suitable for the fabrication of textiles. The spinning solution was subsequently introduced into the instrument, and the spinning process was conducted under the following conditions: a positive spinning voltage of 14 kV, a negative receiving voltage of −3 kV, a spinning distance of 18 cm, injection rate 0.75 mL/h, a temperature control of 30 °C, and a relative humidity maintained at 80%. The obtained fiber membrane was thermostated in a muffle furnace at 200 °C for 2 h to obtain the final sample.

### 2.3. Characterization

An integrated scanning electron microscope-energy spectrometer (SEM/EDS, Phenom ProX, Eindhoven, The Netherlands) was used to observe and measure the surface morphology and elemental distribution of TiO_2_-NaA@PANMs at 10 kV and 15 kV. The functional group characterization of different samples in the experiment was measured and characterized by Fourier transform infrared spectroscopy (FTIR, IRTracer-100, Shimadzu, Kyoto, Japan). The experiments were carried out under a ceramic light source with the speed of the moving mirror set to 2.8 cm/s, the grating delay to 10 s, and 30 cycles to record data in the wavelength range of 400–4000 cm^−1^. The crystal shape of TiO_2_-NaA@PANMs was determined using an X-ray diffractometer (XRD, Rigaku D/MAX 2500V, Rigaku, Tokyo, Japan) with a Cu target, a measurement angle range of 2θ of 10–80°, a scanning speed of 10°/min, and a scanning step of 0.002°. The zeta potential of the samples at different pHs was characterized using a Zetasizer (NanoBrook Omni, Brookhaven, NY, USA). An atomic absorption spectrometer (AAS-7000, NY, USA) was used to determine the concentration of Sr^2+^ in solution. Electron beam irradiation was performed using an electron accelerator (GJ-2-II, Xianfeng electrical plant, Wuxi, China), the electron beam energy was 1.8 MeV, and the variable current was from 0 to 10 mA.

### 2.4. Adsorption Experiment

The effect of condition parameters, such as dosage, pH, adsorption time, initial concentration, dynamic adsorption, cyclic desorption, and irradiation intensity on the removal of Sr^2+^ by TiO_2_-NaA@PANMs, was investigated for S1, where the speed of the shaker was 160 r/min during all adsorption experiments.

### 2.5. Adsorption Parameters

The formulas involved in this experiment are given in the Supporting Materials S2.

## 3. Results and Discussions

### 3.1. Effect of TiO_2_ to PAN Mass Ratio on the Morphology of TiO_2_-NaA@PANMs

**Figure 2:**
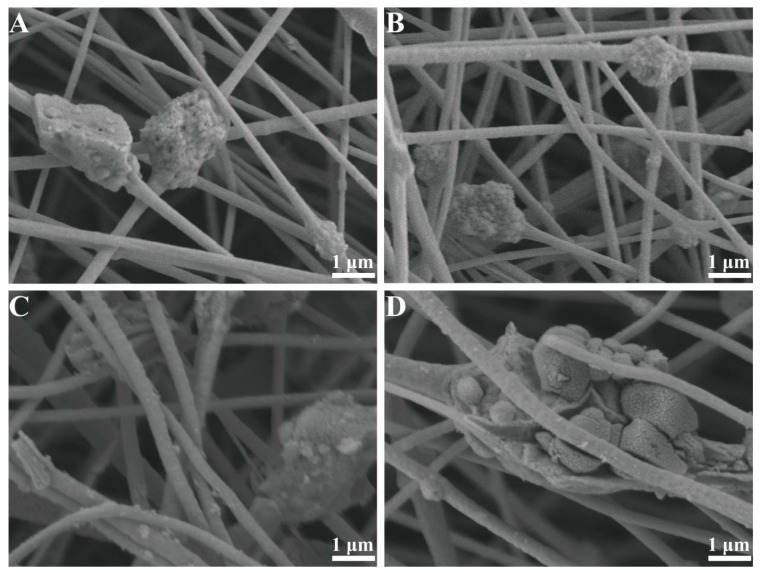
SEM images of 200 °C-TiO_2_-NaA@PANMs with different TiO_2_ to PAN mass ratios (a/b) (**A**) a/b = 2.5%; (**B**) a/b = 5.0%; (**C**) a/b = 7.5%; (**D**) a/b = 10%.

TiO_2_ addition is a key parameter that affects the fiber dimensional morphology. As shown in Figure 2, the TiO_2_ addition concentration significantly affected the dispersion of nanoparticles and the structural integrity of the fibers. When the TiO_2_ concentration was 2.5% (Figure 2A), the particles were sparsely distributed within the fiber matrix, indicating that the nanoparticles failed to fill the polymer network effectively at low concentrations. As the concentration was increased to 5% (Figure 2B), TiO_2_ was uniformly distributed on the PAN fibers, no obvious agglomeration was observed, and the fiber surface was smooth with uniform diameter distribution, indicating good compatibility at the particle–matrix interface at this concentration. However, at concentrations ≥7.5% (Figure 2C), the nanoparticles underwent severe agglomeration, which led to the disruption of fiber continuity and the creation of localized stress concentration areas, which was the key causative factor for the deterioration of the mechanical properties of the samples at high concentrations. In addition, the excess TiO_2_ (10%) significantly changed the rheological properties of the spinning liquid, resulting in the destabilization of the jet during the stretching process in the electric field, which was manifested by the increased filament breakage rate and droplet splashing phenomenon. At the same time, the agglomerates formed tend to block the spinneret holes, resulting in lower continuity of fiber preparation and lower productivity.

### 3.2. Effect of Oxidation Temperature on the Morphology of TiO_2_-NaA@PANMs

**Figure 3:**
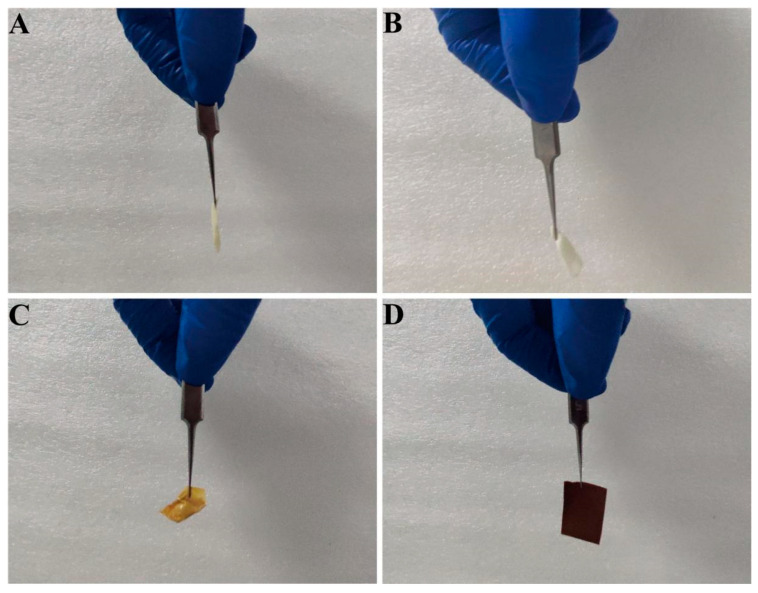
(**A**–**D**) Show the morphology of 50/100/150/200 °C-TiO_2_-NaA@PANMs after adsorption at 75 °C for 2 h in a shaking bed.

In order to further enhance the stability of TiO_2_-NaA@PANMs in water, this paper systematically explores the effect of oxidation temperature on the morphology of fiber membranes. The TiO_2_-NaA@PANMs subjected to varying oxidation temperatures were examined for their topographic stability under water bath conditions at 75 °C. The results are presented in Figure 3. The fiber membranes subjected to treatment at temperatures of 50 °C (Figure 3A) and 100 °C (Figure 3B) exhibited evident signs of deliquescence and agglomeration during the adsorption process. As illustrated in Figure 3C, the fiber membrane that underwent treatment at 150 °C exhibited agglomeration. In contrast, the 200 °C condition (Figure 3D) exhibited consistent performance in the adsorption tests, with no discernible alterations observed before and after the adsorption process. Figure S1 shows that the surface of the fiber membrane is intact and no fracture was found. This finding suggests that the TiO_2_-NaA@PANMs subjected to these temperature conditions possess optimal thermal stability and structural integrity. In summary, the optimal oxidation temperature of 200 °C was determined to be the most effective method for preparing TiO_2_-NaA@PANMs with stable structure and strong temperature adaptability.

### 3.3. XRD and FT-IR Analysis of TiO_2_-NaA@PANMs

**Figure 4:**
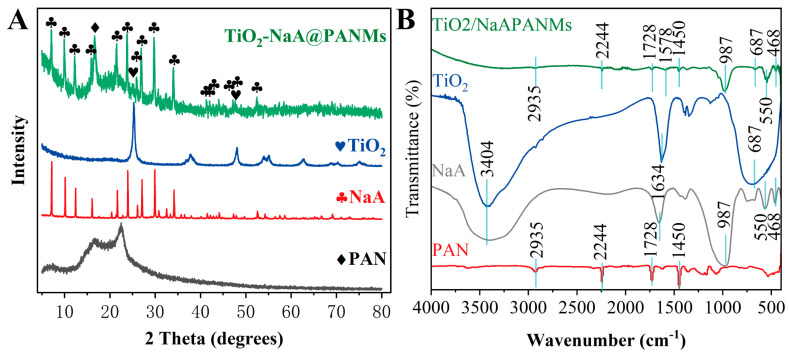
XRD (**A**) and FTIR (**B**) plots of NaA, PAN, TiO_2_, and TiO_2_-NaA@PANMs.

Figure 4A displays the XRD patterns of pristine NaA, pristine PAN, TiO_2_, and TiO_2_-NaA@PANMs. The TiO_2_-NaA@PANMs exhibit broad amorphous peaks corresponding to PAN at 2θ = 16.6° and 22.6°, along with distinct diffraction peaks from NaA and TiO_2_. Notably, no new crystalline phases are detected, indicating that TiO_2_-NaA@PANMs primarily underwent physical blending without chemical reactions. To further elucidate the thermal effects on NaA@PANMs, FTIR analysis of NaA, PAN, TiO_2_, and TiO_2_-NaA@PANMs is presented in Figure 4B. A prominent characteristic peak of NaA zeolite is observed at 987 cm^−1^, attributed to the asymmetric stretching vibration of T-O-Si bonds (T: Si or Al) in the NaA framework. The peak at 468 cm^−1^ arises from the bending vibrations of T-O bonds (T: Si or Al) in the NaA framework [32,33], while the peak at 550 cm^−1^ corresponds to the bending vibration of Si-O-Al bonds within the double four-membered rings (D4Rs) of NaA [34]. Peaks at 1634 cm^−1^ and 3404 cm^−1^ are ascribed to the stretching and bending vibrations of hydroxyl groups (-OH) in adsorbed water molecules. The FTIR spectrum of TiO_2_-NaA@PANMs reveals distinct peaks at 2935 cm^−1^, 2244 cm^−1^, 1728 cm^−1^, and 1450 cm^−1^, which are absent in NaA but align with characteristic peaks of PAN. These peaks are assigned to the stretching vibration of C-H bonds in methyl groups (CH_3_), the stretching vibration of nitrile groups (C≡N), the stretching vibration of carbonyl groups (C=O) from the secondary monomer methyl acrylate in synthesized PAN [35], and the vibration of methylene groups (CH_2_), respectively. Additionally, a hybrid stretching vibration peak at 1578 cm^−1^, distinct from PAN, is observed and attributed to overlapping contributions from C=N, C=C, and O=C-NH bonds. This indicates partial reduction of C≡N groups, accompanied by oxidative dehydrogenation and cyclization reactions in the fibrous membrane. These structural transformations demonstrate that thermal oxidation at elevated temperatures enhances the functional performance of TiO_2_-NaA@PANMs.

### 3.4. Effect of TiO_2_-NaA@PANMs Dosage and pH on Sr^2+^ Adsorption

**Figure 5:**
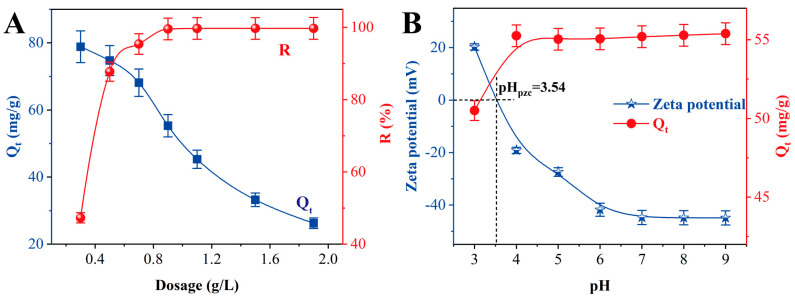
Effect of dosage (**A**) and pH (**B**) on the adsorption of Sr^2+^ by TiO_2_-NaA@PANMs.

In this study, the adsorbent dosing and solution pH for the adsorption performance of Sr^2+^ on thermally modified 200 °C-TiO_2_-NaA@PANMs fiber membranes were systematically investigated. As demonstrated in Figure 5A, the adsorbent dosage was increased from 0.3 g/L to 0.9 g/L, and the Sr^2+^ removal efficiency was increased from 47.30% to 99.7%. Continuing to increase adsorbent mass removes almost the same amount. However, this rise in efficiency was accompanied by a decline in the adsorption capacity per unit mass, from 78.8 mg/g to 26.2 mg/g. This phenomenon can be attributed to the redundancy of adsorption sites triggered by high adsorbent concentration, which resulted in a decrease in adsorption capacity. Specific surface area utilization decreased. A thorough optimization analysis of adsorption efficiency and capacity was conducted, leading to the determination of an optimal adsorbent dosage of 0.9 g/L. Figure 5B illustrates the regulation of adsorption performance by solution pH: at pH = 3, the adsorption capacity of Sr^2+^ was only 50.5 mg/g; the adsorption capacity increased to 55.0 mg/g when the pH was increased to 4; and the adsorption capacity then remained stable in the pH 4–9 range. Zeta potential analysis indicated that the composite’s zero-charge point (pHpzc) was 3.54. When the solution pH was less than 3.54, the surface of the fiber membrane was protonated, forming a positive charge site. Conversely, at pH > 3.54, the negative charge on the surface significantly enhanced the electrostatic attraction. This charge inversion mechanism rationally explains the sudden change in adsorption performance in the pH = 3–4 interval. Given that the pH = 6 of the experimental system Sr^2+^ solution is exactly in the optimal adsorption interval of the material (pH 4–9), this pH condition was selected as the baseline experimental parameter to ensure the reliability and reproducibility of the experimental data. The findings of this study offer a significant theoretical foundation for the optimization of strontium ion adsorption process parameters.

### 3.5. Adsorption Kinetics

**Figure 6:**
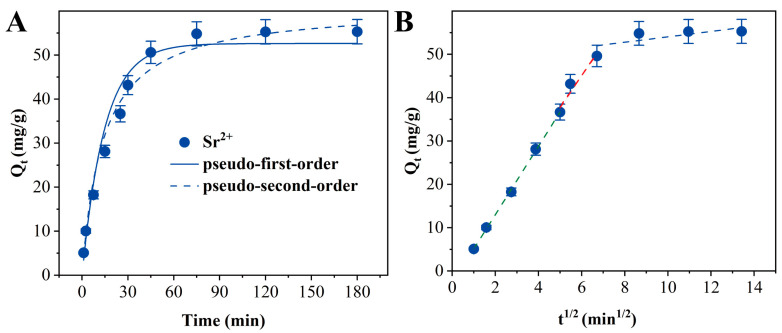
Effect of contact time on the adsorption of Sr^2+^ by TiO_2_-NaA@PANMs and pseudo-first-order and pseudo-second-order kinetic models fitting (**A**), intraparticle diffusion model fitting (**B**).

As illustrated in Figure 6, the adsorption kinetic behavior of Sr^2+^ on 200 °C-TiO_2_-NaA@PANMs can be observed. The experimental data demonstrated a progressive increase in adsorption capacity with time, reaching equilibrium after 45 min. To further resolve this dynamic process, pseudo-first-order and pseudo-second-order kinetic models were used to fit the experimental data. The results are shown in Figure 6A, and the correlation coefficient of the pseudo-second-order kinetic model (R^2^ = 0.968) was significantly higher than that of the pseudo-first-order model (R^2^ = 0.963). The fitting parameters of the different models are shown in Table 1. Additionally, the theoretical equilibrium adsorption capacity (57.08 mg/g) differs from the experimental value (55.00 mg/g) by a mere 3.03%. To further deconstruct the adsorption process, a multi-stage kinetic analysis was carried out by the internal diffusion model. The model fitting results are shown in Figure 6B, which demonstrated that the adsorption process underwent three distinctive phases: (1) liquid film diffusion phase (0–25 min), during which Sr^2+^ migrated rapidly to the surface of the adsorbent through liquid film diffusion; (2) internal diffusion control phase (25–45 min), during which ions were transported to the internal active sites along the pore structure of the material, permeating along the pore structure to reach the internal active sites; this process was synergistically regulated by the pore tortuosity and the concentration gradient; (3) the adsorption–desorption dynamic equilibrium stage, which lasts more than 45 min. In this stage, the surface adsorption sites tend to be saturated, and the adsorption enters into the steady state. The observation that the fitted curves did not pass through the origin suggests that liquid film diffusion is not the sole rate-controlling step and that interfacial mass transfer and intraparticle diffusion collectively influence the adsorption kinetic behavior. This study systematically elucidated the time-varying characteristics and mass-transfer rate-limiting mechanism of Sr^2+^ adsorption on 200 °C-TiO_2_-NaA@PANMs by employing a multi-modeling strategy.

### 3.6. Adsorption Isotherm

**Figure 7:**
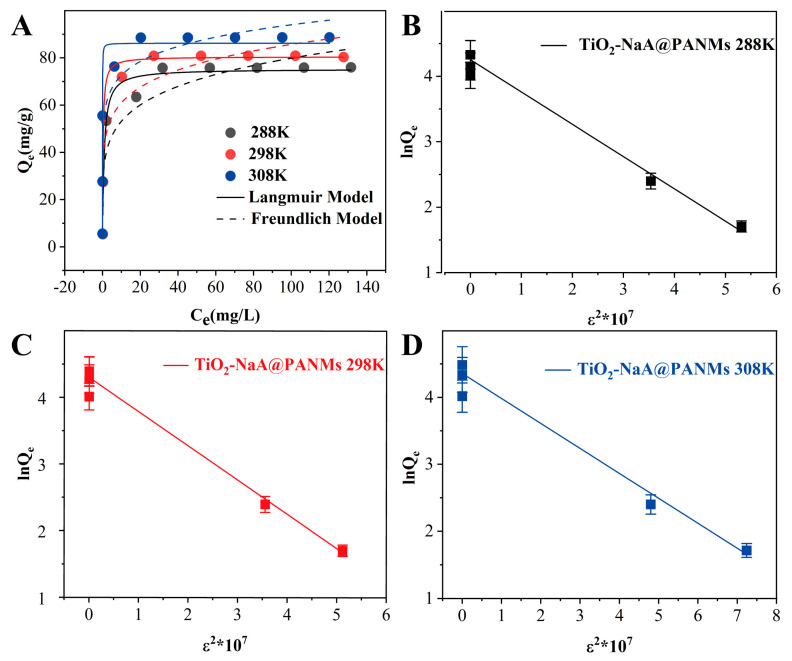
Effect of temperature and initial concentration on the adsorption of Sr^2+^ by TiO_2_-NaA@PANMs and fitting plots for Langmuir, Freundlich (**A**) and D-R (**B**–**D**) model.

The equilibrium adsorption capacity of TiO_2_-NaA@PANMs on Sr^2+^ at varying initial concentrations and temperatures is demonstrated in Figure 7A. It is evident from the figure that the equilibrium adsorption capacity exhibited a substantial increase with an increase in Sr^2+^ concentration from 5 mg/L to 75 mg/L. When the initial Sr^2+^ concentration was less than 50 mg/L, the removal efficiency of TiO_2_-NaA@PANMs on Sr^2+^ reached 99% or higher. However, when the initial concentration was increased from 75 mg/L to 200 mg/L, the adsorption on the fiber membrane tended to reach a state of saturation. This was due to the lack of sufficient active sites, which prevented further adsorption of the remaining Sr^2+^. The maximum saturated adsorption capacity exhibited a gradual increase in response to an elevation in temperature from 288 K to 298 K and 308 K, ranging from 75.76 mg/g to 80.89 mg/g and 88.56 mg/g, respectively. To thoroughly investigate the intrinsic mechanism of the adsorption process, the Langmuir, Freundlich, and Dubinin-Radushkevich (D-R) models were employed to fit the adsorption data at varying temperatures. The resulting fitting curves are presented in Figure 7, and the relevant parameters are summarized in Table 2. The adsorption behaviors of TiO_2_-NaA@PANMs on Sr^2+^ were consistent with the Langmuir model, and the maximum saturated adsorption capacities measured experimentally at different temperatures were in agreement with the theoretical value of 75.30 mg/g. Theoretical values of 75.30 mg/g, 80.49 mg/g, and 86.22 mg/g are highly correlated with the maximum saturated adsorption capacity of TiO_2_-NaA@PANMs at different temperatures was approximately 75.76 mg/g, 80.89 mg/g, and 88.56 mg/g, providing substantial evidence that supports the adsorption mechanism of the monomolecular layer and directly verifies the promotion of the adsorption process by warming. Figure 7B–D was analyzed using a D-R model, and the thermodynamic functions were exhaustively analyzed by the van’t Hoff isothermal equations. Key thermodynamic parameters such as Gibbs free energy (ΔG^θ^), enthalpy change (ΔH^θ^), and entropy change (ΔS^θ^) were also computed and are shown in Table 3. The decrease in the ΔG° value with the increase in temperature from 288 K to 308 K reconfirms that an increase in temperature facilitates the adsorption process. Concurrently, the positive values of both the enthalpy of adsorption (ΔH^θ^) and the entropy of adsorption (ΔS^θ^), in conjunction with the negative value of the free energy of adsorption (ΔG^θ^), collectively indicate a spontaneous process of heat absorption. This phenomenon is further elucidated by the accelerated diffusion rate of ions in solution at elevated temperatures, thereby promoting enhanced adsorption performance. Table 4 provides comparative data between TiO_2_-NaA@PANMs and other Sr^2+^ adsorbent materials in terms of maximum adsorption capacity.

### 3.7. Dynamic Adsorption

**Figure 8:**
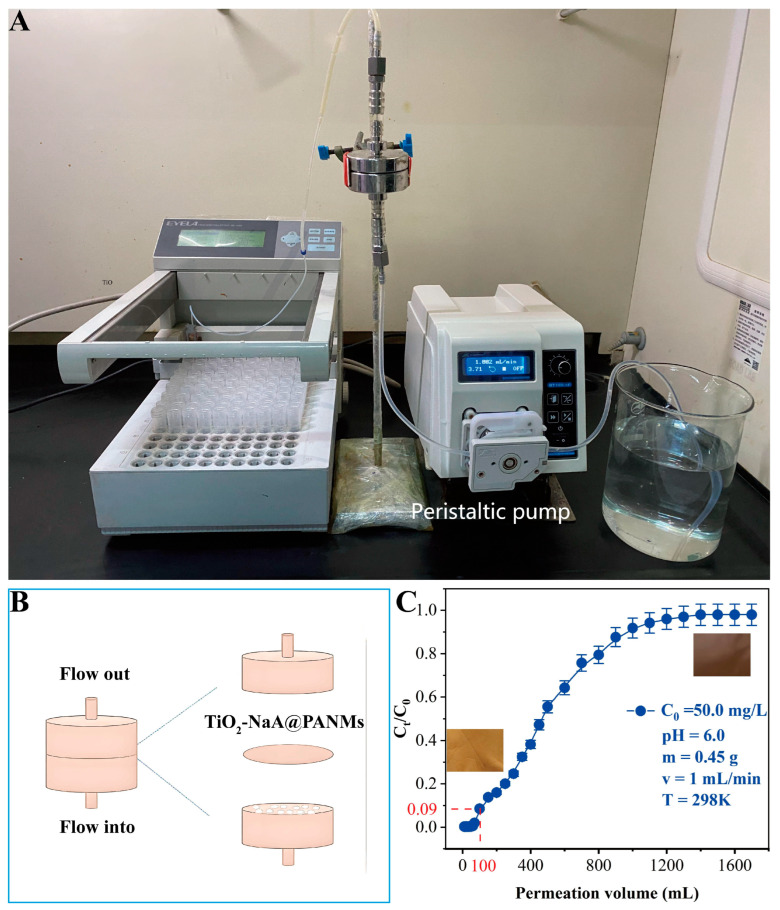
The experimental apparatus employed for the dynamic removal of Sr^2+^ is depicted in (**A**); an enlarged view of the membrane device is shown in (**B**); the dynamic adsorption of Sr^2+^ by TiO_2_-NaA@PANMs is demonstrated in (**C**), with the inset depicting the optical images corresponding to the initial state (left) and the final state (right) of the dynamic adsorption of TiO_2_-NaA@PANMs.

The dynamic adsorption experiments are a valuable tool for assessing the practical application performance of membrane materials. The experimental setup is illustrated in Figure 8A,B. The assembly of TiO_2_-NaA@PANMs with a diameter of 5 cm was conducted within a membrane filtration device, and the Sr^2+^ solution was introduced into the filtration device by means of a peristaltic pump. The adsorption permeation curve is a reflection of the dynamic adsorption process of the adsorbent, and the concentration ratio of the adsorbent to the initial time of the effluent to the permeate volume after adsorption is calculated using Equations (1) and (2) to obtain the amount of Sr^2+^ at saturation. m_e_ is the adsorbed amount of Sr^2+^ at saturation (mg), and M_Tot_ is the total mass of Sr^2+^ in the solution. S is the total mass of unadsorbed Sr^2+^. The integral area between the curve in the permeation curve and the axis, qe, is the dynamic adsorption capacity (mg/g), and m is the mass of the membrane (mg). Assembled in the membrane filtration unit, the Sr^2+^ solution was pumped into the filtration cell via a peristaltic pump at a flow rate of 1.0 mL/min. The adsorption capacity is a useful parameter to demonstrate the adsorption efficiency, as shown in Figure 8C. The dynamic adsorption process had the capacity to manage 100 mL of feed solution, and the dynamic adsorption capacity of TiO_2_-NaA@PANMs for Sr^2+^ during the dynamic adsorption process was calculated to be 55.56 mg/g. The optical images of the nanofiber membranes remained largely unchanged after the dynamic adsorption, thereby confirming the stability of the nanofiber membranes within the dynamic system.

m_e_ = m_Total_ − S (1)

q_e_ = m_e_/m (2)

### 3.8. Desorption and Circulation

**Figure 9:**
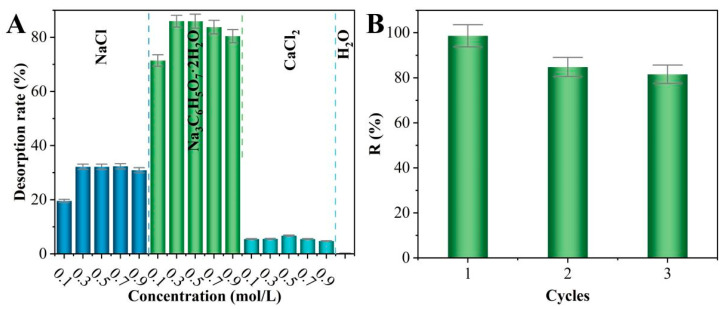
The desorption effect of various desorbents and concentrations on TiO_2_-NaA@PANMs (**A**); the impact of the number of cycles on Sr^2+^ removal (**B**).

The cyclic regeneration performance of TiO_2_-NaA@PANMs is also a crucial measure when exploring the potential of TiO_2_-NaA@PANMs for industrial applications. To this end, the desorption efficiency of the material was initially investigated with different desorbing agents as well as at different concentrations. As demonstrated in Figure 9A, TiO_2_-NaA@PANMs exhibited minimal desorption in a pure water environment. Conversely, the desorption rate of 0.3 mol/L sodium citrate solution-TiO_2_-NaA@PANMs reached 85.97%, which was notably higher than that of NaCl (25.55%) and CaCl2 (6.7%) solutions. Consequently, 0.3 mol/L sodium citrate was selected as the optimal desorbent for the adsorption–desorption cycle experiment. The cycling effect is demonstrated in Figure 9B, and the removal rate persists at 81.60% even after three cycles, signifying that TiO_2_-NaA@PANMs exhibits notable cycling efficacy for Sr^2+^ and possesses potential for reuse in industrial applications.

### 3.9. Effect of Irradiation on the Adsorption Properties of TiO_2_-NaA@PANMs

**Figure 10:**
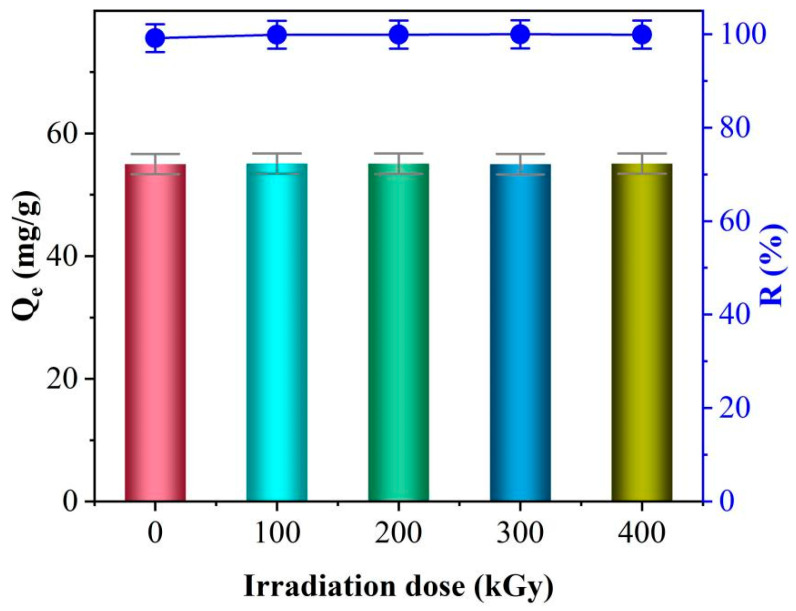
Effect of irradiation dose on the removal of Sr^2+^ by TiO_2_-NaA@PANMs. The blue line represents the removal rate, and the bar chart represents the adsorption capacity.

In order to more accurately evaluate the potential of TiO_2_-NaA@PANMs for Sr^2+^ adsorption under the radiation environment, in this study, the TiO_2_-NaA@PANMs were exposed to four distinct doses of gamma rays, ranging from 100 kGy to 400 kGy. The TiO_2_-NaA@PANMs were subjected to adsorption experiments, and the results are shown in Figure 10, where the irradiation-treated fibrous membranes were used to adsorb the Sr^2+^ solution with an initial concentration of 50 mg/L. With the initial concentration of Sr^2+^ maintained constant, the adsorption capacity and removal rate of Sr^2+^ by TiO_2_-NaA@PANMs exhibited no decline as the irradiation dose increased from 100 kGy to 400 kGy. This finding suggests that TiO_2_-NaA@PANMs possess considerable potential for the removal of radioactive strontium elements from nuclear wastewater.

## 4. Conclusions

In this study, NaA zeolites were prepared by a simple non-hydrothermal method, and TiO_2_-NaA@PANMs were successfully synthesized by a combination of electrostatic spinning and oxidation method with a controlled injection rate of 0.75 mL/h at TiO_2_:NaA:PAN = 1:20:20 and a pre-oxidation temperature of 200 °C, which combined the ion exchange capacity of NaA molecular sieves and the radiation stability of nano- and nano-TiO_2_, providing an innovative solution for the selective scavenging of Sr^2+^ in complex aqueous environments. TiO_2_, which combines the ion-exchange capacity of NaA molecular sieves and the radiation stability of nano-TiO_2_, provides an innovative solution for the selective scavenging of Sr^2+^ in complex aqueous environments. In the static adsorption experiments of Sr^2+^, the adsorption equilibrium could be rapidly adsorbed within 60 min at pH = 6 and at a mass of 0.9 g/L of TiO_2_-NaA@PANMs, and the removal rate was as high as 99.0%. The adsorption process conformed to the quasi-second-order kinetic model and the Langmuir model, the adsorption reaction was a spontaneous adsorption process, and increasing the temperature was favorable for the adsorption reaction. The adsorption effect was stable after irradiation at 400 kGy. Meanwhile, TiO_2_-NaA@PANMs also had a good dynamic separation effect. Under the conditions of flow rate of 1 mL/min and mass of 0.45 g, the dynamic adsorption amount of Sr^2+^ by TiO_2_-NaA@PANMs in the dynamic adsorption process was calculated to be 55.56 mg/g, and the dynamic adsorption process could process 100 mL of feed solution. This material will provide a new solution to the problem of nuclear wastewater treatment and help promote the further development of related technologies.

## Figures and Tables

**Table 1:** Kinetic parameters of Sr^2+^ adsorption by TiO_2_-NaA@PANMs.

		Pseudo-First-Order			Pseudo-Second-Order			IPD		
Ion Type	Q_e,exp_	Q_e,cal_	K_1_ (×10^−4^)	R^2^	Q_e,cal_	K_2_ (×10^−4^)	R^2^	K_id_	C	R^2^
Sr^2+^	55.00	52.62	676.7	0.963	57.08	12.8	0.979	7.41	0.65	0.968

Q_e,exp_ and Q_e,cal_ (mg/g); K_1_ and K_2_ (×10^−4^); K_id_ (mg/(g·min^1/2^)).

**Table 2:** Isotherm model fitting results for Sr^2+^ adsorption by TiO_2_-NaA@PANMs at temperature and initial concentration.

Isotherm Model	Parameter	Temperature (K)		
		288	298	308
Langmuir	Q_e,exp_	75.76	80.89	88.56
	Q_max_	75.30	80.49	86.22
	K_L_	1.20	3.41	49.17
	R^2^	0.958	0.934	0.910
Freundlich	n	5.66	6.77	8.92
	K_F_	35.40	43.43	56.12
	R^2^	0.820	0.757	0.824
D-R	Q_m_	73.78	78.07	82.76
	β (×10^−6^)	4.93	5.13	3.73
	E	0.35	0.36	0.27
	R^2^	0.986	0.991	0.959

Q_e,exp_ and Q_max_ (mg/g); K_L_ (L/mg) and K_F_ ((mg/g)/(mg/L)^1/n^); β (mol^2^/J^2^) and E (kJ/mol).

**Table 3:** Thermodynamic parameters of Sr^2+^ adsorption by TiO_2_-NaA@PANMs.

Temperature (K)	Thermodynamic Parameters		
	ΔG^θ^ (kJ·mol^−1^)	ΔH^θ^ (kJ·mol^−1^)	ΔS^θ^ (J·mol^−1^·K^−1^)
288	−2.15	23.1	87.0
298	−2.70		
308	−3.76		

**Table 4:** Comparison of the adsorption capacity of TiO_2_-NaA@PANMs with membrane materials reported in the literature.

Adsorbing Material	Dosage	pH	Maximum Adsorbing Capacity	Reference
▪ PSA5	1.00 g/L	6	45.80 mg/g	[35]
▪ BCM@APTES-EDTA	1.00 g/L	6	44.86 mg/g	[36]
▪ SA-PA-H	1.00 g/L	6	151.7 mg/g	[37]
▪ KTS@PAN	1.00 g/L	6	32.4 mg/g	[38]
▪ GO/Ni-MOF	0.40 g/L	7	72.00 mg/g	[39]
▪ PVA/GO/MnO_2_	0.40 g/L	7	28.81 mg/g	[40]
NaA@PANMs	0.90 g/L	6	80.89 mg/g	This work

Ref. [35]: Polyvinyl alcohol hydrogel membranes were prepared by crosslinking with sulfosuccinic acid in different ratios (5% relative to the PVA monomer). Ref. [36]: Bacterial cellulose membrane (BCM) modified with ethylenediaminetetraacetic acid (EDTA) using (3-aminopropyl) triethoxysilane (APTES) as a crosslinker. Ref. [37]: a thiol-rich carboxyethyl grafted pentaerythritol tetrakis (thioglycolic acid) ester (PA) synthesized by click chemistry was used to covalently crosslink the hydrogel (SA-PA-Sr) with abundant thiol groups simultaneously introduced and lastly, a Sr^2+^-imprinted adsorbent (SA-PA-H). Ref. [38]: potassium tin sulfide (KTS-3) was dispersed evenly and fixed to the polyacrylonitrile (PAN) support. Ref. [39]: a novel self-assembled membrane consisting of metal–organic framework (MOF) nanobelts and graphene oxides (GOs) are synthesized through a simple and facile filtration method. Ref. [40]: Polyvinyl alcohol (PVA) and Mn^2+^ played a synergistic role in the gelation of PVA/GO/Mn^2+^, while Mn^2+^ can be further converted into oxide to achieve functionalized aerogel (PVA/GO/MnO_2_).

## Data Availability

The original contributions presented in this study are included in the article and Supplementary Materials. Further inquiries can be directed to the corresponding authors.

## Supplementary Materials

The following supporting information can be downloaded at: https://www.mdpi.com/article/10.3390/ma18092151/s1, S1: Adsorption experimental conditions; S2: Adsorption parameters and formulae; S3: SEM images; Figure S1: SEM images of 200 °C-TiO_2_-NaA@PANMs after adsorption at 75 °C for 2 h in a shaking bed.; Table S1: Adsorption experimental conditions. Refs. [41,42,43] have been cited in Supplementary Materials file.
