# Supplementary material for: Preparation of NaA Zeolite Composite Polyacrylonitrile Membranes (TiO2-NaA@PANMs) Doped with TiO2 and Adsorption Study of Sr2+
Source: Materials (Basel). 2025 May 7;18(9):2151. doi: 10.3390/ma18092151 (PMC12072940; doi:10.3390/ma18092151)
Supplement: Supplementary file 1 [file materials-18-02151-s001.zip › materials-3597748-supplementary.pdf]

Supplementary Material

Preparation of NaA Zeolite Composite Polyacrylonitrile Membranes (TiO_2-NaA@PANMs) Doped with TiO_2 and Adsorption Study of Sr^{2+}

Yu Liu[1], Riwen Ji[2]*, Kaituo Wang[1]*

[1]School of Resources, Environment and Materials, State Key Laboratory of Featured Metal Materials and Life-cycle Safety for Composite Structures, MOE Key Laboratory of New Processing Technology for Nonferrous Metals and Materials, Guangxi Key Laboratory of Processing for Non-ferrous Metals and Featured Materials, Guangxi University, Nanning 530004 Guangxi, P. R. China

[2]Guangxi Zhuang Autonomous Region Institute of Product Quality Inspection, Nanning 530007, China

***Corresponding author's** e-mail: jiriwen@163.com; wangkaituo@gxu.edu.cn

S1 Adsorption experimental conditions

Table S1. Adsorption experimental conditions

Condition parameters	Operation process
**Dosage**	Dosage was optimized in a range of 0.3-1.8 g/L using 50 mg/L Sr^{2+} solution and pH=6 for 2 h at 25 °C constant temperature water bath oscillator.
**pH**	pH was optimized in the range of 3-9 at dosage of 0.9 g/L with 50mg/L Sr^{2+} solution for 2 h at 25 °C constant temperature water bath oscillator.
**Adsorption time**	Adsorption time was optimized in a range of 1-180 min using dosage of 0.9 g/L for Sr^{2+} solution (pH=6, 50 mg/L) at 25 °C constant temperature water bath oscillator.
**Initial concentration**	Initial concentration was optimized in a range of 5-200 mg/L at dosage of 0.9 g/L for Sr^{2+} for 2 h at pH=6 in 15, 25 and 35 °C constant temperature water bath oscillator.

**Dynamic adsorption**	Sr^{2+} solution at a concentration of 50 mg/L was pumped through a peristaltic pump at a flow rate of 1 mL/min into a filtration device equipped with a 0.45 g fiber membrane, and the concentration of Sr^{2+} in the effluent solution was measured and recorded by sampling every 10 mL.
**Desorption and circulation**	A range of concentrations of NaCl, $CaCl_2$, sodium citrate, and deionised water were utilised as desorbents for the desorption experiments, which were conducted for a duration of 120 minutes. The Sr^{2+} concentration was evaluated and documented. The fiber membrane resolved by sodium citrate was re-adsorbed with a Sr^{2+} solution at a concentration of 50 mg/L. This was then tested and recorded, followed by desorption for 2 h, adsorption for 2 h, and the process was repeated three times consecutively.
**Effect of irradiation on TiO_2-NaA@PANMs**	Following the irradiation of TiO_2-NaA@PANMs with an electron beam, four samples were collected at irradiation doses of 100 kGy, 200 kGy, 300 kGy, and 400 kGy, respectively. A total of 0.027 g of TiO_2-NaA@PANMs was weighed and added to 30 mL of a Sr^{2+} solution with an initial concentration of 50 mg/L for a 120 min adsorption period. This study aimed to investigate the impact of irradiation dose on the adsorption performance of TiO_2-NaA@PANMs.

S2 Adsorption parameters and formulae

In this experiment, the removal rate (R) and adsorption capacity (Q_e) were calculated by Eq.1(1) and Eq.2(2) [1]:

$$R(\%)=\frac{C_0-C_e}{C_0}\times 100 \tag{1}$$

$$Q_e=\frac{V(C_0-C_e)}{m} \tag{2}$$

Where C_0 and C_e (mg/L) are the initial and equilibrium concentration, respectively, V (L) is the volume of the solution, and m (g) is the mass of the adsorbent.

In this experiment, the experimental data of adsorption time will be fitted by the pseudo-first-order and pseudo-second-order dynamics model as Eq.3 (3)and Eq.4 (4)

[2]:

$$Q_t = Q_e\left(1-e^{-k_1 t}\right) \quad (3)$$

$$Q_t = \frac{Q_e^2 k_2 t}{1+Q_e k_2 t} \quad (4)$$

Here, Q_t and Q_e (mg/g) are the adsorbed amount at adsorption time t (min) and at equilibrium, respectively. And k_1 (min^{-1}) and k_2 ($g\cdot mg^{-1}\cdot min^{-1}$) are the rate constants for pseudo-first-order and pseudo-second-order, respectively.

The velocity control step was explored using the intraparticle diffusion model of Eq.5(5):

$$Q_t = k_i t^{\frac{1}{2}} + C_i \quad (5)$$

Where k_i ($mg/(g\cdot min^{1/2})$) is the diffusion constant, C_i represents the thickness of the liquid film.

Isothermal adsorption data were fitted by the Langmuir model of Eq.6 (6), the Freundlich model of Eq.7(7) and the D-R model of Eq.8 (8), Eq.9(9) and Eq.10 (10), respectively [3]:

$$Q_e = \frac{Q_m K_L C_e}{1+K_L C_e} \quad (6)$$

$$Q_e = K_F C_e^{\frac{1}{n}} \quad (7)$$

$$\ln Q_e = \ln Q_m - \beta\varepsilon^2 \quad (8)$$

$$\varepsilon = RT\ln\left(1+\frac{1}{C_e}\right) \quad (9)$$

$$E = \frac{1}{\sqrt{2\beta}} \quad (10)$$

Where Q_m (mg/g) is the maximum adsorption capacity at equilibrium, K_L (L/mg) is the equilibrium constant of Langmuir model; K_F ($(mg/g)/(mg/L)^{1/n}$) and n are the adsorption equilibrium constants of Freundlich isotherm model. β (mol^2/J^2) is the adsorption energy constant, ε (kJ/mol) is the Polanyi potential energy, R (8.314 $J\cdot mol^{-1}\cdot K^{-1}$) is the general gas constant and T (K) is the absolute temperature. E (kJ/mol)

shows the adsorption free energy.

The parameters of the adsorption thermodynamic function are calculated as shown in Eq.11 **Error! Reference source not found.**, Eq.12 **Error! Reference source not found.**and Eq.13**Error! Reference source not found.**, respectively [4]:

$$K_{\mathrm{d}} = \frac{Q_e}{C_e} \tag{11}$$

$$\Delta G = \Delta H - T\Delta S \tag{12}$$

$$lnK_d = \frac{\Delta S}{R} - \frac{\Delta H}{RT} \tag{13}$$

Where R (8.314 J·mol^{-1}·K^{-1}) is the general gas constant and T (K) is the absolute temperature, K_d is the equilibrium constant that is dimensionless. It is calculated by converting the unit of K_L (the best isotherm model fitted) to mol/L using the molecular weight of the adsorbate, ΔG (kJ·mol^{-1}) represents Gibbs free energy, ΔH (kJ·mol^{-1}) represents enthalpy change of reaction, ΔS (J·mol^{-1}·K^{-1}) represents entropy change of reaction.

S3 SEM images

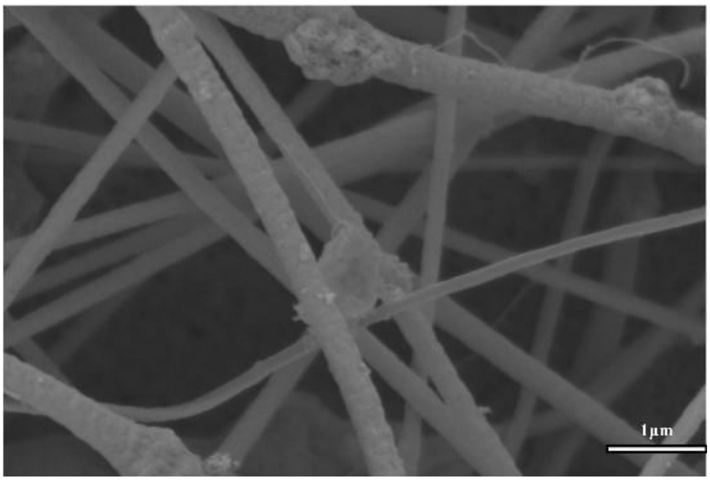

Fig.S1. SEM images of 200 °C-TiO_2-NaA@PANMs after adsorption at 75 °C for 2 h in a shaking bed.

References

[1] Yi, M., Wang, K., Wei, H., et al., 2023. Efficient preparation of red mud-based geopolymer microspheres (RM@GMs) and adsorption of fluoride ions in wastewater, J. Hazard. Mater., 442,

130027. DOI: https://doi.org/10.1016/j.jhazmat.2022.130027.

[2] Wang, K., Wang, F., Chen, F., et al., 2019. One-Pot preparation of NaA zeolite microspheres for highly selective and continuous removal of Sr(II) from aqueous solution, ACS Sustain. Chem. Eng., 7, 2459-2470. DOI: https://doi.org/10.1021/acssuschemeng.8b05349.

[3] Lei, H., Muhammad, Y., Wang, K., et al., 2021. Facile fabrication of metakaolin/slag-based zeolite microspheres (M/SZMs) geopolymer for the efficient remediation of Cs^{+} and Sr^{2+} from aqueous media, J. Hazard. Mater., 406, 124292. DOI: https://doi.org/10.1016/j.jhazmat.2020.124292.

[4] Wang, K., Chen, S., Qiu, R., et al., 2022. Convenient preparation of activated carbon modified phosphoric acid-activated geopolymer microspheres (C@PAAGMs) for the efficient adsorption of ReO_4^-: Implications for TcO_4^- elimination, Compos. Part B-Eng., 247, 110296. DOI: https://doi.org/10.1016/j.compositesb.2022.110296.
